# Smart fertigation: Automated fertilizer application based on plant growth using digital sensors with IoT capability

**DOI:** 10.1371/journal.pone.0357482

**Published:** 2026-09-03

**Authors:** Sangrak Son, Jin Wei-Kocsis, Amanda Deering, Lori Hoagland, Krishna Nemali

**Affiliations:** 1 Horticulture and Landscape Architecture, Purdue University, West Lafayette, Indiana, United States of America; 2 Polytechnic Institute, Purdue University, West Lafayette, Indiana, United States of America; 3 Food Science, Purdue University, West Lafayette, Indiana, United States of America; Institute of Genetics and Developmental Biology Chinese Academy of Sciences, CHINA

## Abstract

Liquid organic fertilizers (LOF) are used to supply additional nutrients to crops. However, LOF are expensive to use regularly. In this research, a smart fertigation treatment (S), where LOF was applied based on lettuce plant growth, was compared to a manual treatment (M) in a greenhouse. Digital sensors with IoT capability were installed in S and M treatments. The sensor in S treatment was interfaced to a solid-state relay connected to a pump, to automatically regulate LOF application. Digital sensors in both treatments measured canopy area (CA) of plants once every three days using image analysis and remotely exchanged data. In the M treatment, LOF was applied every third day to plants, whereas the LOF application was regulated by the sensor in S treatment to happen only when CA of plants was lower by 10% than those in the M treatment. Results indicated no differences in fresh weight (33.4 vs. 32.3 g·plant^−1^) and leaf area (770.2 and 772.6 cm^−2^) of plants between two treatments. Nutrient concentration in the root zone, measured as electrical conductivity of substrate, was significantly higher (21–69%) in M than S treatment. The number of fertigation events and cost of LOF applied were approximately four times higher in M than S treatment. This resulted in higher fertilizer-use-efficiency in S (1.159 kg ∙ m^−2^ ∙ $^−1^) than M (0.168 kg ∙ m^−2^ ∙ $^−1^) treatment. These results indicate that the smart fertigation method can optimize LOF application (i.e., reduce wastage and costs) while maintaining crop growth.

## Introduction

Organic fertilizers incorporated into the substrate generally contain low concentration of nutrients [1]. Liquid organic fertilizers (LOF) can be added to the substrate for increasing nutrient supply to organically grown crops in greenhouses. The LOF are made of organic materials such as agricultural manures and fish byproducts [1,2]. During manufacturing processes, LOF undergoes enzymatic hydrolysis, extraction, anaerobic digestion, and fermentation that break down organic materials and increase the concentration of readily available nutrients [3–5]. Organic nutrients in LOF are in a stable condition during storage as further microbial activity is minimized by low pH (typically pH 3–5) or thermal processes [6–9]. The LOF can be applied at any time during production [10,11]. However, the main drawback of using LOF is their high cost ($ 6–7 L^−1^) [12] leading to an increase in the overall cost of production.

Generally, liquid fertilizers are supplied to plants at pre-determined concentration or electrical conductivity (EC) level [13,14]. In some instances, the EC of the root zone (EC_sub_) is measured [15,16] to determine the quantity and timing of liquid fertilizer application. These methods are intended to maintain enough nutrients in the root zone. However, mere presence of nutrients in the root zone may not always directly influence plant growth. The nutrients consumed by plants should be used in metabolism for plant growth to happen [17]. In many instances, plants tend to ‘luxuriously’ consume nutrients than what is needed for metabolism [18], and store them in vacuoles [19–21].

A better approach is to supply liquid fertilizers based on plant growth. This method involves initially applying a small volume of liquid fertilizer and holding off next application until a measurable reduction in plant growth is observed, due to the lower concentration of nutrients in the substrate and plant tissue. This method not only ensures that most of the nutrients in the root zone are absorbed but also utilized by plants in growth processes. Further, it is likely that ‘luxury’ consumption is minimal as nutrients are not supplied in excess. For this method, it is important to accurately, continuously, and non-invasively monitor plant growth to ensure that growth reductions are small and temporal.

Digital sensors can capture images of plants for continuous and non-invasive estimation of canopy area (CA, a two-dimensional estimate of total leaf area), a surrogate for plant growth [22,23]. Relative changes in CA were closely related to relative growth rate of plants based on biomass measurements [22]. Low-cost microcontrollers (e.g., Raspberry Pi, Arduino) can be interfaced with camera modules and programmed to continuously capture plant images, process the images using image analysis software (e.g., Python/ OpenCV), and estimate canopy area of plants [22,24]. These microcontrollers can be programmed to work like IoT (internet of things) devices for enabling remote communication and data exchange [24]. Further, these systems can be connected to electronic controllers (e.g., solenoids or relays) to control pumps and automatically deliver liquid fertilizer to plants. Such smart fertigation systems can significantly reduce liquid fertilizer use and decrease production related costs not only in organic production but in conventional greenhouse production. However, the efficacy of such digital systems for automated nutrient delivery to plants based on plant growth was never tested in greenhouse production.

In this research, we tested the efficacy of a novel and custom-built smart fertigation system that supplied LOF to plants based on plant growth. To the best of our knowledge, research on greenhouse grown crops with similar smart fertigation technology based on plant growth was never tested before. The objectives of this study were to (i) develop a smart fertigation system that utilizes digital sensors to continuously monitor plant growth, enables communication and decision support on IoT, and supplies fertilizer automatically based on plant growth and (ii) test the efficacy of the developed smart fertigation system to optimize LOF application by comparing with a manual method of application.

## Materials and Methods

### Plant materials and Crop production

The experiment was conducted for 21 days in a glass greenhouse. Organic seeds of buttercrunch lettuce (*Lactuca sativa* cv. ‘Rex’) were purchased (Johnny’s Selected Seeds, Winslow, ME) and sown in a peat-based germination medium (BM2, Berger, QC, Canada) using 72-cell seed trays (54 cm × 27 cm × 3 cm; Greenhouse Megastore, Danville, IL). After sowing, the trays were placed in a mist irrigation zone to ensure uniform germination. Ten days after sowing, seedlings were transplanted into square plastic pots (9.3 cm × 9.3 cm × 7.9 cm; Greenhouse Megastore, Danville, IL) filled with a custom prepared soilless substrate composed of sphagnum peat moss (Sun Gro Horticulture, Agawam, MA, USA) and vermiculite (Oldcastle Lawn and Garden, Atlanta, GA, USA) in a 4:1 (peat moss: vermiculite, v/v) ratio. A custom prepared organic fertilizer (Table 1) was incorporated into the substrate prior to filling in the pots. Each pot contained approximately 4.6 g of substrate incorporated organic fertilizer. The environmental conditions to which plants were exposed during the experiment include an average temperature of 21.5 ± 2.69 °C, average humidity of 82.5 ± 21.16%, and average daily light integral of 9.8 ± 1.32 mol·m^−2^·day^−1^. The pH of the substrate ranged between 5.7 ± 0.14 and 5.9 ± 0.24. The EC of the substrate varied with treatments (see below).

**Table 1:** List of fertilizers used in preparing customized substrate-incorporated organic fertilizer mix, and their approximate weight in each pot.

Fertilizer Components	Weight (mg/pot)	Manufacturer
7-5-7	3333	California Organic Fertilizer Inc.
4-3-2	830	D. Stutzman Farms
Epsom Salt	250	Lawn and Garden Products Inc
Ferrous Sulfate	83	Brandt Consolidated Inc
Manganese Sulfate	8	Brandt Consolidated Inc
Fertibor	42	U.S. Borax Inc.
Total	4546	

### Irrigation system

Plants were grown in a custom-built automated recycling sub-irrigation system utilizing constant flood tables (CFT; 121.9 cm × 30.5 cm × 8.9 cm; Botanicare, Vancouver, WA) placed on greenhouse benches (762 cm × 150 cm × 110 cm). Each CFT tray was connected to a reservoir (76 L; Active Aqua Premium, Petaluma, CA) to form a closed-loop recycling irrigation system. The reservoir was filled with approximately 40 L of reverse osmosis (RO) water to ensure that no additional nutrients were added to plants through irrigation water. The inlet of the CFT tray was connected to a submersible pump (530 L ∙ hr^−1^; Total Pond, West Palm Beach, FL) that delivered water to the tray via vinyl tubing (1.6 cm ID, (Crop King Inc., Lodi, OH). The pump was operated for 15 minutes each day for sub-irrigation, which maintained sufficient moisture for the plants in the substrate. Flow valves (Green Back in-line valve; Botanicare) were attached to the inlet tubing to maintain a constant water flow. A short vertical pipe (5 cm) was inserted into the outlet hole of the CFT tray to control the level of water that accumulates in the tray before draining back to the reservoir. When pumps were stopped, any remaining water in the CFT tray drained back into the reservoir through the inlet tubing.

### Treatments

**Fig 1:**
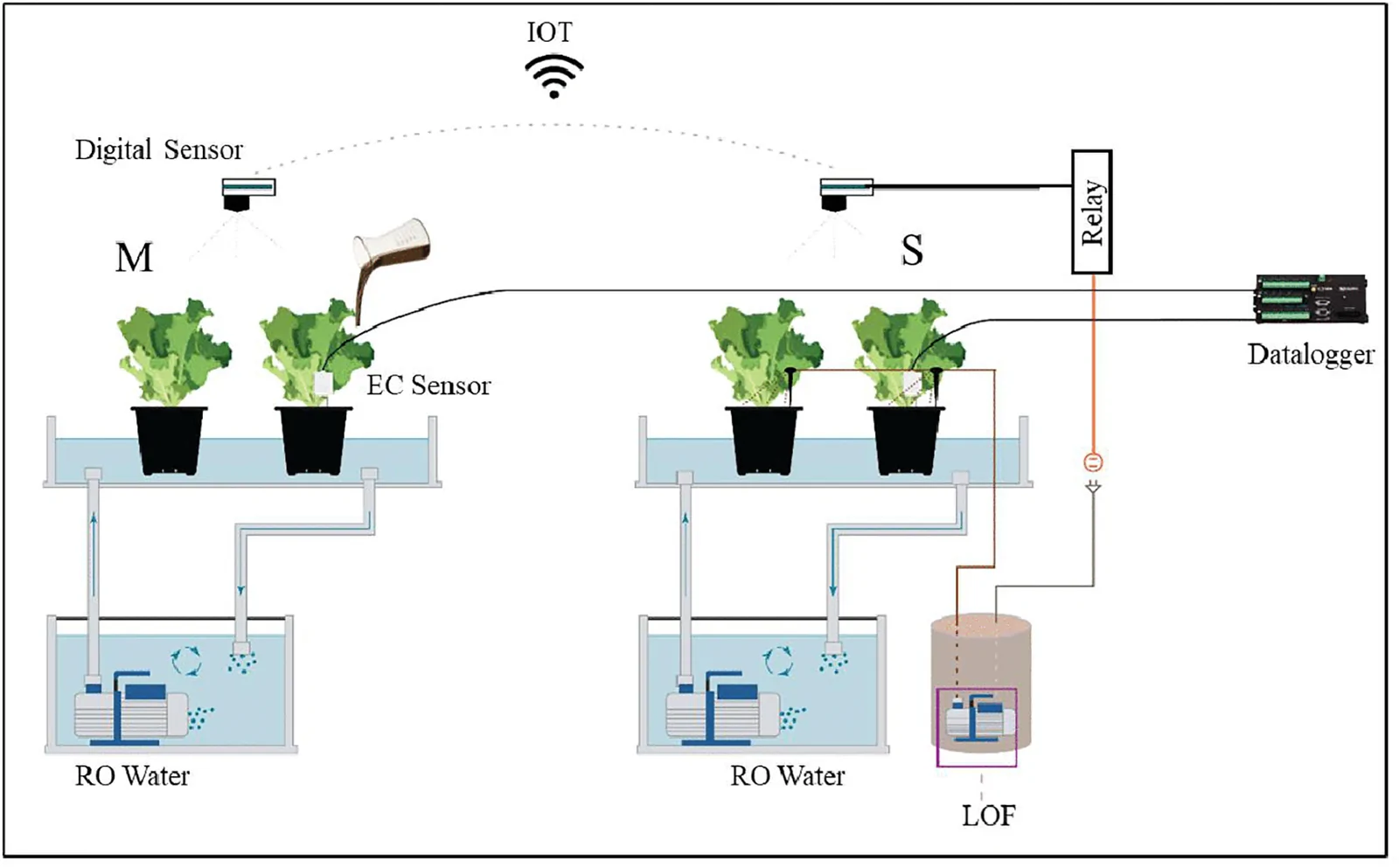
An illustration of manual (M) and smart fertigation (S) based treatments. The reverse osmosis water was stored in reservoirs. The liquid organic fertilizer (LOF) was manually supplied to plants every 3^rd^ day in the M treatment. The application of LOF was based on the decision made by the digital sensor, in the S treatment. The digital sensor closed a relay when the canopy area of plants in S treatment was lower than that of M treatment by 10%, turned on a submersible pump and supplied LOF to plants for a given duration.

The experiment used LOF containing 3% N, 3% P_2_O_5_, and 2% K_2_O (AgroThrive Inc., Gonzales, CA). The manufacturer supplied LOF was diluted by mixing with RO water at a concentration of 10 mL ∙ L^−1^ before applying to plants. There were two treatments of LOF applications to plants (Fig 1), including manual (M) and smart fertigation (S). In the M treatment, LOF was supplied manually once every three days to plants. A total of six LOF applications, each with 50 mL ∙ pot^−1^, were applied to plants in this treatment during the study. The LOF was applied uniformly on top of the substrate in each pot. Plants in the S treatment received LOF automatically based on the decision made by the smart fertigation system (see below for details). In each fertigation, plants in the S treatment also received 50 mL ∙ pot^−1^ of LOF.

### Smart fertigation system

This was developed using a combination of digital sensors, solid-state relays, software for IoT communication and decision support, and drip-based fertigation system. Both M and S treatments contained a digital sensor (Fig 1) which was custom-built using a microcontroller (Raspberry Pi 3B, Cana Kit Corp., OR) interfaced with a camera module (Raspberry Pi Camera V2.0, 8 MP, Cana Kit Corp.). The digital sensor was placed above the plants to capture images once every three days and estimate CA using built-in Python-OpenCV based image processing software. The sensors were programmed to exchange CA information between the M and S treatments using IoT. The digital sensor in the S treatment was connected to a solid-state relay (Relay, SONGLE relay, SRD-05VDC-SL-C, Amazon USA) in the normally open (NO) position (i.e., circuit does not allow electricity to flow through relay). The CA measurements by the digital sensors in M and S treatments were remotely communicated with each other once every three days using IoT. The digital sensor in the S treatment compared CA measurements and closed the relay (or energized the pump) when the CA in the S was lower than that in M by 10% or more. When images of plants are taken continuously, it is possible that small changes in leaf angle due to ambient lighting conditions can affect canopy area measurements. A threshold of 10% was chosen to ensure that a real change in canopy area happened between two measurements. The cable supplying power to the submersible pump for LOF application was connected to the relay interfaced with the digital sensor (Fig 1). Because the digital sensor imaged the plants once every three days, a decision to close the relay was made at three-day intervals. This allowed the detection of definitive changes in canopy area in response to a previous fertigation event.

In the S treatment, LOF was stored in separate 40 L reservoirs and applied to the pots using a drip method (Fig 1). A submersible pump (530 L ∙ hr^−1^, Total Pond, West Palm Beach, FL) connected to vinyl tubing (1.6 cm ID, Crop King Inc., Lodi, OH) was placed in the reservoir containing the LOF. Pressure-compensated drip emitters were inserted into vinyl tubing at regular intervals. The emitters were connected to drip stakes (MUF-SPK4, Rain Bird Corporation, Tucson, AZ) by capillary tubing (5 mm, Netafim^TM^, Fresno, CA). One drip stake was inserted at the center of each pot. When the submersible pump was turned on, the LOF was supplied to the substrate as a cone of spray from the drip stake. The volume of LOF applied was maintained by running the pumps for a pre-determined duration.

### Measurements

Both M and S treatments contained reflectometers (CS655; Campbell Scientific, Logan, UT) connected to a datalogger (CR1000, Campbell Sci.) to continuously monitor EC_sub_ (dS ∙ m^−1^) every hour and store daily averages. Percentage difference in EC_sub_ was calculated between M and S treatments for any given day. In addition, change in EC_sub_ was calculated at three-day intervals, i.e., start and end of each LOF application in M treatment and corresponding times in S treatment. The CA (cm^2^) of plants was automatically estimated at three-day intervals and stored in the microcontroller.

The number of fertigation events were 6 (twice per week and for three weeks) in the M treatment, whereas they were automatically recorded by the digital sensor in each replication of the S treatment. From this number, the total volume of LOF supplied to plants was calculated by multiplying total fertigation events with application volume (50 mL ∙ pot^−1^) in each event. The total volume of concentrated LOF used was calculated by multiplying the total volume of LOF applied with the dilution rate (10 mL ∙ L^−1^). The total volume of concentrated LOF used per plant was adjusted to m^2^ area (mL·m^−2^) by multiplying it with 43 plants∙m^−2^. The cost of LOF ($ ∙ m^−2^) used was calculated by multiplying volume of concentrated LOF with $6 L^−1^. The total cost of fertilizer ($ ∙ m^−2^) was calculated by adding the cost of substrate incorporated fertilizer to the cost of concentrated LOF. For calculations, $0.39 m^−2^ was used as the cost of substrate-incorporated organic fertilizer (based on purchase cost of fertilizers).

At the harvest stage, the pH of the substrate was measured by collecting the leachate and using a pH sensor (Groline, Hannah Instruments, RI). Fresh biomass of shoots (FW, g∙plant^−1^) was measured by weighting the shoots that were cut at the ground level. Total leaf area (cm^2^) was measured by separating leaves and running them through the rollers of a leaf area meter (LI-3100C, LI-COR Environmental, Lincoln, NE). The shoot material was placed in a paper bag and dried in a forced air oven maintained at 70°C for a week to determine the dry weight (DW, g∙plant^−1^). Fertilizer use efficiency (FUE, kg ∙ $^−1^) was calculated by dividing the FW of plants∙m^−2^ area (i.e., g∙plant^−1^ × 10^−3^ kg ∙ g^−1^ × 43 plants∙m^−2^) with the total cost of fertilizer applied ($ ∙ m^−2^).

### Statistical analysis

The experiment was laid out in a randomized complete block design with two fertilizer treatments and four replications. Data were analyzed using a mixed model (“Proc Mixed”) procedure of statistical analysis software (SAS, version 9.4, SAS Institute, Cary, NC, USA). Repeated measures analysis was used for CA and EC_sub_ measurements, which were measured on same plants every third day and every day, respectively. The means were compared using Tukey’s honestly significant difference (HSD) test at a significance level of *P* ≤ 0.05. All graphs were created using Microsoft Excel software.

## Results

### Biomass and leaf area

The average FW was not statistically different between M (33.4 g·plant^−1^) and S (32.3 g·plant^−1^) treatments (Table 2). Similar responses were observed for DW (1.47 vs 1.43 g·plant^−1^) differences between the fertilizer treatments (Table 2). There were no significant differences between the two fertilizer treatments on total leaf area of plants (Table 2). The average leaf area of plants in M and S treatments was 770.2 and 772.6 cm^−2^, respectively. There was a linear relation between FW and LA (FW = 0.042 ∙ LA, *r*^*2*^ = 0.99; data not shown), when data were pooled from both fertilizer treatments.

**Table 2:** Effect of fertilizer treatment on shoot fresh weight (FW, g·plant^−1^), shoot dry weight (DW, g·plant^−1^), and leaf area (LA, cm^2^) of lettuce. Fertilizer treatments were manual (M) and smart fertigation (S) methods of liquid organic fertilizer application. Least square means with standard error of the mean in parenthesis are shown. Means with same letters are not statistically different (*P* < 0.05) between fertilizer treatments.

Fertilizer	FW	DW	LA
	(g·plant^−1^)	(g·plant^−1^)	(cm^2^·plant^−1^)
M	33.4 (3.99) a	1.47 (0.258) a	770.2 (84.89) a
S	32.3 (3.46) a	1.43 (0.235) a	772.6 (83.89) a

### Canopy area

**Fig 2:**
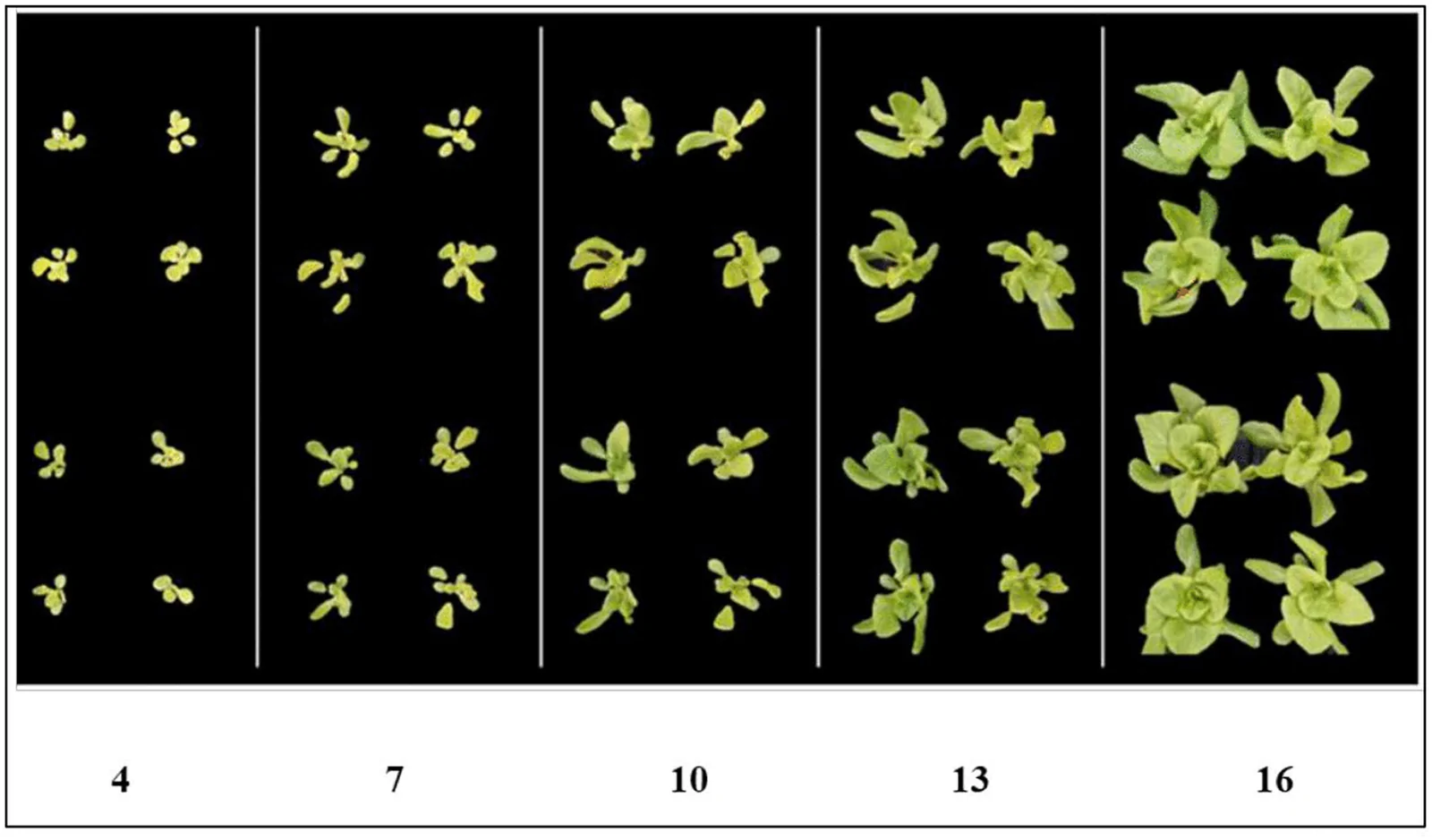
An illustration of segmented images of lettuce plants in the manual (M, top row) and smart (S, bottom row) fertigation treatments on different days of the experiment. The images were taken by the digital sensor every 3^rd^ day during growth and processed using custom image analysis software and calibration to segment the background and estimate the canopy area of plants.

Image analysis software accurately estimated CA by effectively segmenting green leaves from the background (Fig 2). There was a minimal canopy overlap among different plants on any given day within a treatment. The image analysis software did not estimate the area of the leaves shaded by upper leaves and include it in CA estimation. Visual images of plants showed that CA increased slowly during the first 10 days and rapidly increased during the last six days of the study.

**Fig 3:**
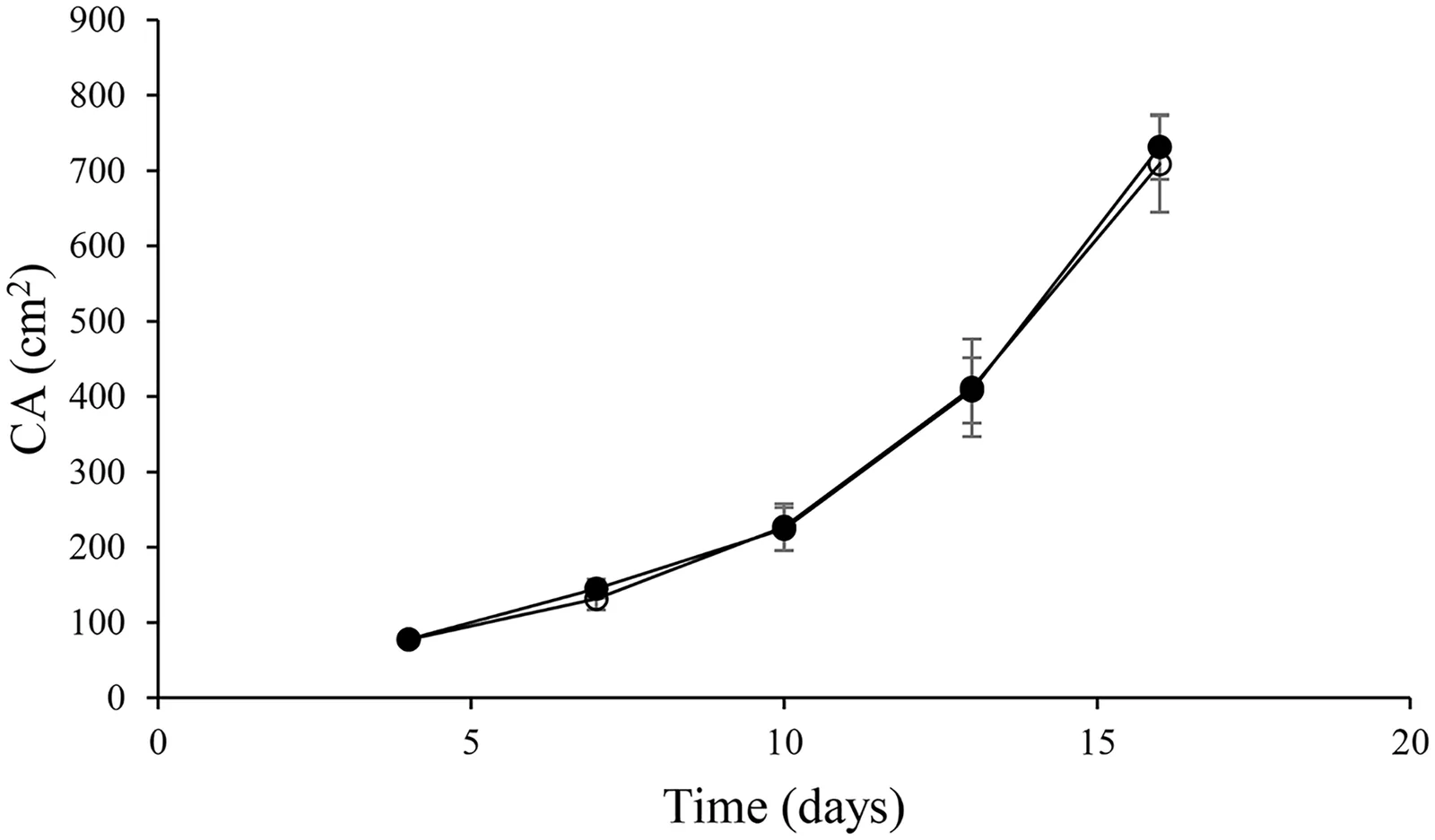
Change in canopy area (CA, cm^−2^) of lettuce with time in the manual (M, black circles) and smart fertigation (S, open circles) treatments. The CA was measured every third day starting from day 4 of the study. Each data point represents the average of four replications. The least-square means error bars indicating the standard error of the mean are shown for both treatments.

The main effect of time was significant (*P* < .0001), whereas the effect of fertilizer treatment was not significant (*P* = 0.505) on CA (Fig 3). This indicates that CA increase with time was different (expected), but the increase with time was not different between M and S treatments. In both treatments, CA increased slowly up to day 10 (on an average, 15 cm^2^ ∙ d^−1^) and beyond this it increased rapidly (on an average, 82 cm^2^ ∙ d^−1^) during the remaining measurement period. A numerically higher canopy area in M than S treatment was observed on days 7 and 16 of the study.

### Electrical conductivity of substrate

**Fig 4:**
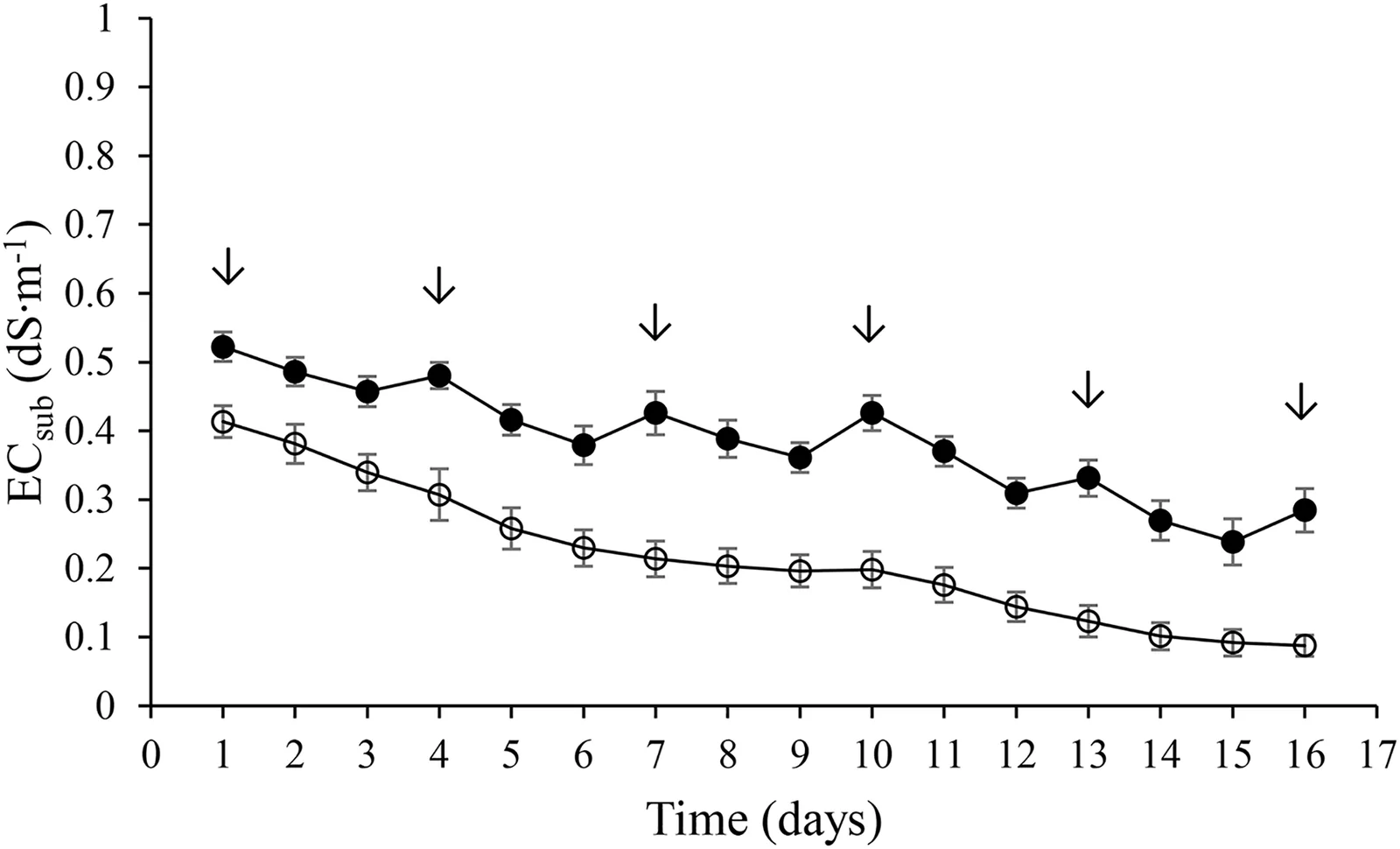
Interactive effect of fertilizer treatment and time on substrate electrical conductivity (EC_sub_, dS·m^−1^). Fertilizer treatments are manual (M, black circles) and smart fertigation (S, open circles) methods of liquid organic fertilizer (LOF) application. The EC_sub_ was measured for 16 days during growth. Arrows represent days on which M treatment received LOF application. Each data point represents average of 24 measurements per day and four replications. The least-square means with standard error of the mean are shown.

The interaction between fertilizer treatment and time was significant (*P* < 0.0001) for EC_sub_ (Fig 4), indicating that fertilizer treatment effects on EC_sub_ varied with measurement time (day). On day 1, the LOF application in M resulted in higher EC_sub_ compared to that of S. The value of EC_sub_ in both M and S treatments decreased with time. However, the rate of decrease was slower in M than in S leading to larger differences between treatments with time. The EC_sub_ was higher in M than S throughout the measurement period. The difference in EC_sub_ between M and S treatments on day 1 was approximately 21 (after LOF application) and increased continuously up to 69% by day 16 in S (Fig 4).

**Fig 5:**
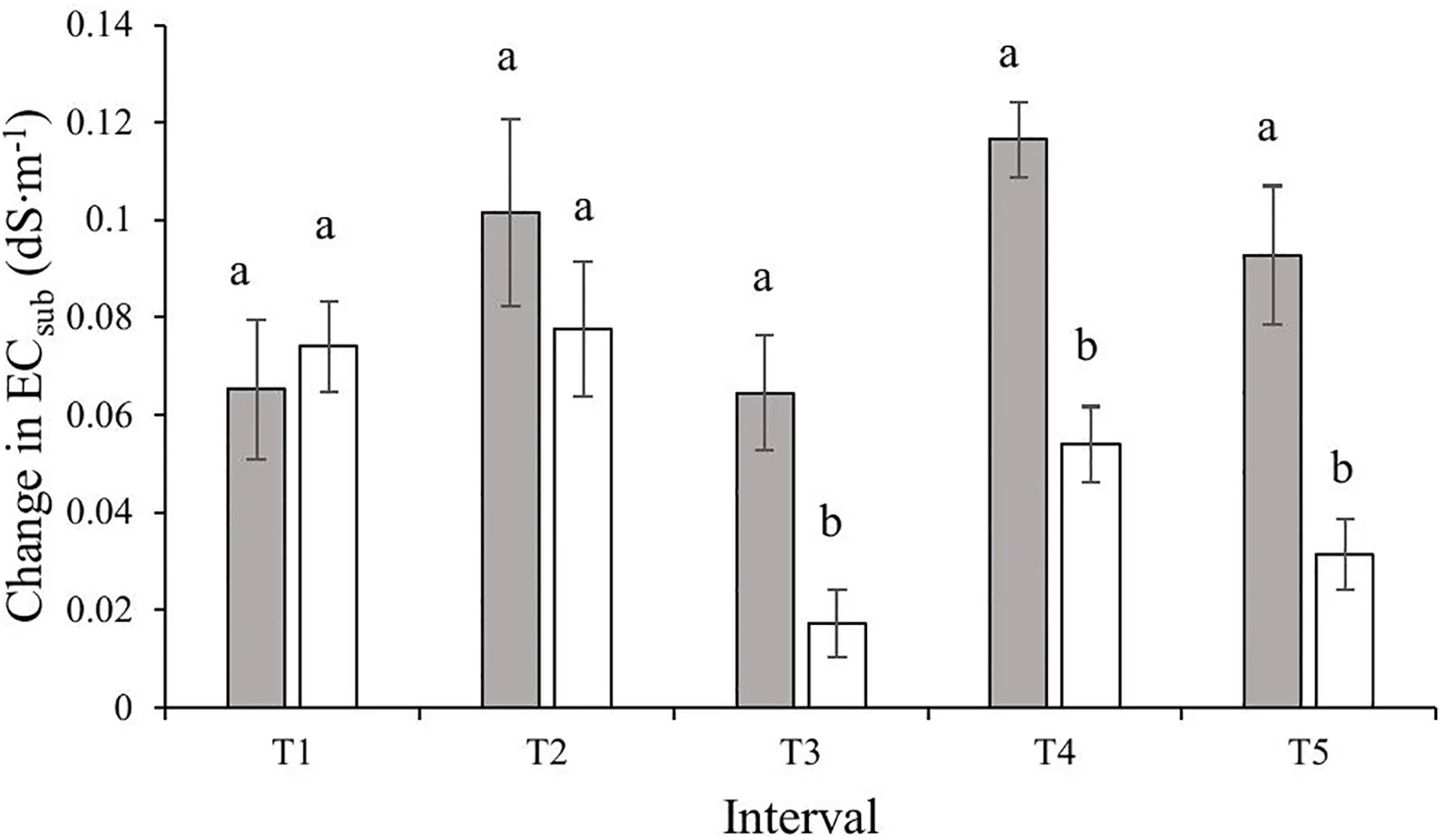
Change in electrical conductivity of substrate (EC_sub_, dS·m^−1^) during three-day intervals in the manual (M, gray bars) and smart fertigation (S, white bars) treatment. Intervals T1, T2, T3, T4, and T5 correspond to days 1-3, 4-6, 7-9, 10-12, and 13-15 respectively. Each bar represents average of four replications. The least-square means with standard error of the mean are shown.

The change in EC_sub_ measured at three-day intervals (Fig 5) was not different between M and S treatments up to T2 stage (or 6 days of treatment imposition). However, the change in EC_sub_ was significantly higher during T3 to T5 stages (from day 7 to day 15) in M compared to S treatment. During these three stages, the change in EC_sub_ between start and end of a fertigation cycle was two to three folds greater in M than S treatment.

### Fertigation and fertilizer-use-efficiency

The average number of fertigation events and volume of LOF application in M and S treatments was significantly different (*P* < 0.0001). In the M treatment, plants were fertigated more times (on an average, 6.0 vs 1.5 events) and received more LOF volume (on an average, 199.0 vs 32.2 mL ∙ m^−2^) than in S treatment (Table 3). The number of fertigation events in the S treatment varied among different replications (note the SE of the mean in Table 3 and S1 Table) due to differences in plant growth among replications. The overall cost of fertilizer, including the cost of substrate incorporated fertilizer and LOF, in M and S treatments was significantly different (Table 3). The cost of fertilizer was higher in M ($8.53 m^−2^) than that in S ($2.43 m^−2^) treatment. Fertilizer treatment significantly affected FUE in plants (Table 3). The FUE in S treatment (1.158 kg·$^−1^) was significantly higher than M treatment (0.168 kg**·**$^−1^). Because of differences in plant growth, a larger variability was also observed for FUE among replications (Table 3).

**Table 3:** Fertigation events, volume of liquid organic fertilizer (LOF) applied, total (substrate incorporated plus LOF) cost of fertilizer, and fertilizer-use-efficiency (FUE) in the manual (M) and smart fertigation (S) treatments. The data was converted to m^2^ area by multiplying with 43 (number of plants m^−2^). Least square means with standard error of mean (in parenthesis) are shown. Means with different letters are statistically different.

Fertilizer Treatment	Fertigation Events	LOF Volume	Total Cost	FUE
		(mL·m ^− 2^)	($·m ^− 2^)	(kg ∙ $ ^− 1^)
M	6.0 (±0.00) a	129.0 (±0.00) a	8.53 a	0.168 (0.0202) b
S	1.5 (±1.00) b	32.2(±21.50) b	2.43 b	1.159 (0.6303) a

## Discussion

We developed a smart fertigation system that can apply liquid fertilizer based on plant growth as opposed to EC measurements. The developed smart fertigation system was tested for its efficacy to save liquid fertilizer and reduce fertilizer cost compared to a manual method of fertilizer application. A critical component of the smart fertigation system is the digital sensor with IoT capability and decision support. The digital sensors were programmed to capture images of plants in the early morning (7 am) to ensure high image quality. Bright light falling on the canopy can interfere with image processing, especially with segmentation (i.e., separating background from leaves). Due to the low solar angle in the early morning, the images did not get exposed to bright light and the image segmentation of the digital sensor was accurate (Fig 2). Further, the estimated CA and actual LA were within a close range (Table 2, Fig 3) in our study. This indicates that most of the leaf area was captured in the images and segmentation of the leaves was achieved with little error. In our study, images of four plants were used to measure the average CA in each experimental unit, to increase the accuracy of measurement (Fig 2). The digital sensor was able to accurately capture CA from early growth stages (i.e., from day 4 after transplanting, Fig 2) when leaf area was small. The changes in CA over time showed an exponential growth pattern (Fig 3), which is expected in many crops including lettuce [21,25].

**Fig 6:**
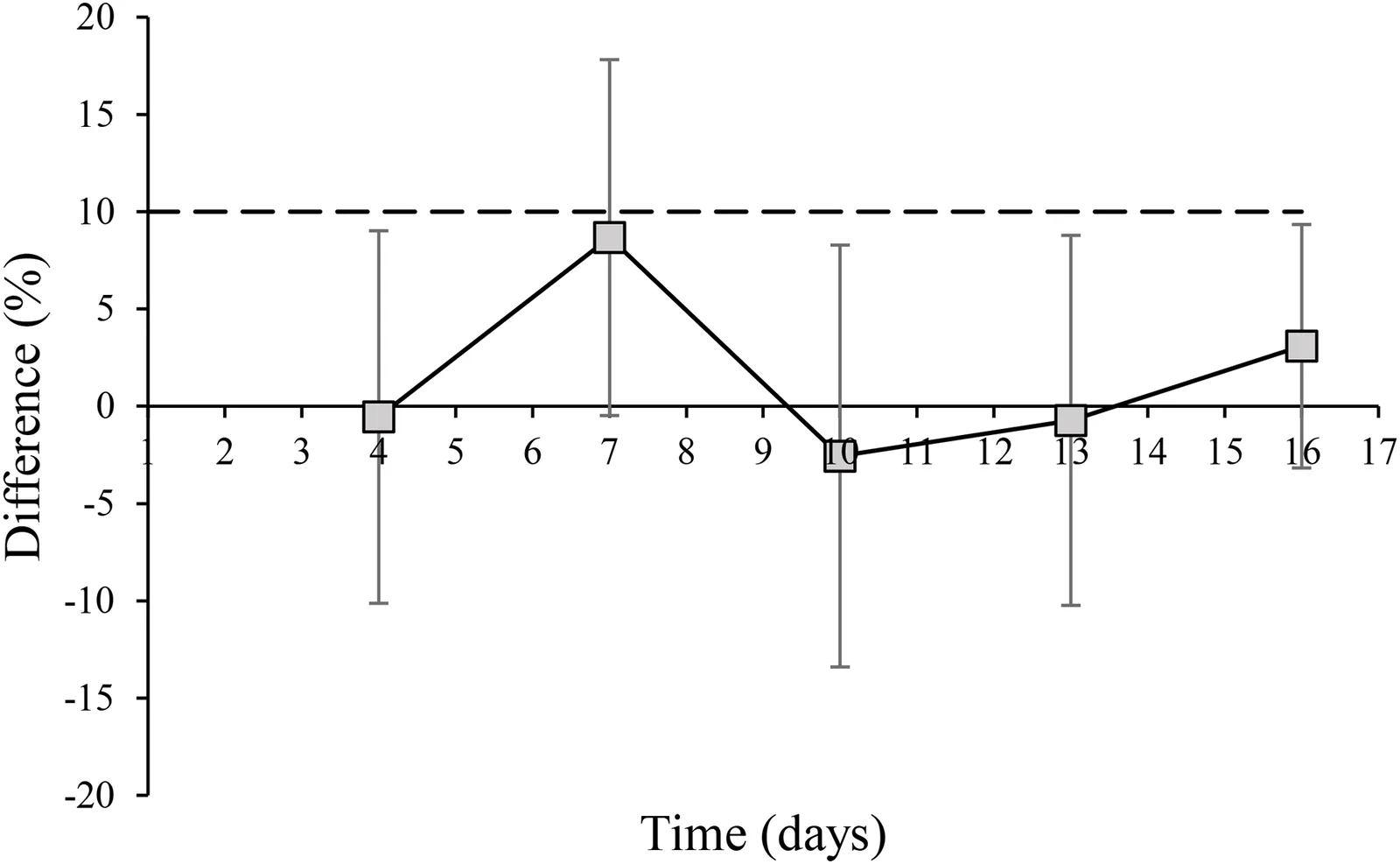
Percent difference in canopy area (CA) between manual (M) and smart fertigation (S) treatments on different days of the experiment. The dashed line represents the target difference above which the electronic relay in S treatment was programmed to close to trigger fertigation. Each data represents average of four replications. The least-square means with standard error of the mean are shown.

The smart fertigation system tightly regulated fertilizer application. The fertigation events happened only when the CA was lower than the targeted 10% in the S than M treatment. Averaged across replications, the percentage difference in CA between M and S treatments was at or above the targeted 10% difference around day 7 (Fig 6; S2 Table). On other days, there were little differences in CA between M and S treatments. The LOF addition that happened around 7 days after transplanting in the S treatment likely minimized differences in CA between treatments by promoting leaf growth in S. As the difference in CA was reduced to below the targeted 10% (Fig 6; S2 Table), the need for additional LOF application was absent during most of the days. This indicates that LOF application happened selectively according to plant demand in the S compared to M treatment. Because of this, the total fertigation events, LOF application volume, and associated fertilizer costs were significantly lower in the S than M treatment (Table 3). The reduced cost of fertigation in the S ($6.10 m^−2^, Table 3) translates to savings of approximately $25,000 per acre (as 1 acre = 4046 m^2^) compared to M treatment. These results suggest that the cost of LOF can be reduced in greenhouses using smart fertigation method.

The relation between biomass and leaf area observed in our study was previously shown for lettuce and other crops [26,27]. Leaf growth supports increased light interception and photosynthesis leading to increased biomass accumulation in plants [26,28]. As CA is a surrogate measure of LA [29], the estimate was used to regulate the LOF application based on plant growth in our study. No differences observed in CA (Fig 3) also aligns with absence of statistically significant differences in FW, DW, and LA between M and S treatments (Table 2). This indicates that CA based regulation is an effective strategy to maintain plant growth due to the relation with LA. The higher FUE observed in S than M treatment (Table 3) is due to maintenance of CA and plant growth (or biomass) while lowering LOF application volume and costs in the S treatment (Tables 2 and 3). Based on our results, smart fertigation systems have the potential to maintain plant growth while reducing wastage of LOF.

The EC sensor measurements were sensitive to small changes observed in our study. Generally, EC_sub_ values are lower in organic production as nutrient concentration in the substrate incorporated organic fertilizer is low [30]. The EC measurements accurately detected increase in EC_sub_ on the day of LOF application and gradual decrease during the next two days in the manual treatment (Fig 4). The sensor measures bulk EC, which is generally lower than the commonly measured pore water EC measurements [16]. The EC_sub_ was higher in the M than S treatment every day of the study (Fig 5) due to the continuous application of LOF in the M treatment. The higher EC_sub_ in the M treatment (Fig 4) indicates increased concentration of nutrients in the substrate in the M compared to S treatment. Although nutrient levels in plants were not measured in the study, a significant and higher change in EC_sub_ in the M than S treatment (Fig 5) suggests an increased uptake of nutrients by plants in this treatment (note that plants were sub-irrigated where leaching is practically absent). On the other hand, a significantly lower change in EC_sub_ in S than M treatment was observed during the corresponding period (Fig 5).

The increased nutrient availability in M than S treatment did not result in plant growth differences. This may be associated with ‘luxury consumption’ of nutrients; however, we could not validate it by measuring tissue nutrient levels. Many crop species exhibit ‘luxury consumption’ when nutrient concentration is high in the substrate [18,31–33]. These nutrients are not immediately used in biomass production. They are usually stored in vacuoles for future needs [34,35]. Leafy greens with high levels of nitrates stored in vacuoles when consumed can pose health issues in humans [36,37]. For this reason, it is a general recommendation to reduce the fertilizer supply to hydroponically grown crops at the end of production cycle for decreasing tissue nitrate levels [38,39]. Although we did not measure nutrients accumulated in the tissue in M and S treatments, it is possible that relatively lower levels of nutrients supplied to plants in S treatment may have resulted in lower levels of nitrates in plants than those in M treatment.

Our research was limited to one variety of lettuce and conducted only for a duration of three weeks. However, the results from our research are compelling and suggest the potential value of growth-based fertigation in reducing wastage of liquid fertilizer. Smart fertigation technology is not affected by crop species or variety but only by differences in crop growth. Therefore, it is expected to work regardless of species or variety. Further, longer duration studies will likely result only in larger fertilizer gains from smart fertigation technology.

Our research is likely the first one to demonstrate the benefits of automatically supplying fertilizer using remote plant growth measurements based on digital sensors in greenhouse crop production. This method of production can significantly advance fertilizer application technology in greenhouses over applications based on both EC of in-line fertilizer solution or substrate. Our research shows that timed fertilizer applications can potentially result in excess application of nutrients without significant growth benefits. The timed fertigation method may increase nutrient availability to plants. However, mere presence of nutrients in the substrate may not warranty plant nutrient uptake as factors such as substrate pH can influence solubility of nutrients in the root zone [40]. In addition, increased uptake may not necessarily result in increased plant growth [41]. Fertilizer applications based on plant growth ensure that nutrients are used optimally by plants. When adopted in large scale, the smart fertigation method has the potential to reduce imports of critical fertilizers, such as nitrogenous and phosphorus fertilizers [42,43], thereby contributing to economic growth.

## Conclusions

We demonstrated the efficacy of the smart fertigation system in *Lactuca sativa* cv. ‘Rex’ in a greenhouse experiment conducted for 21 days. Efficacy was demonstrated using three criteria including (i) accuracy of data collection, (ii) capability to reduce wastage of LOF application, and (iii) capacity to maintain plant growth, compared to a manual application treatment. The smart fertigation system supplied LOF based on canopy growth and plant demand for nutrients as opposed to differences in nutrient status of the substrate. This technique can optimize LOF application in organic lettuce production leading to lower fertilizer costs. Although the system was tested in one cultivar for a short duration using LOF in the present study, it has potential applications for other crops and conventional fertigation application in greenhouses. The system can potentially reduce fertilizer wastage and environmental pollution from fertilizer leaching and run off. Future studies should test the efficacy of the smart sensor system in conventional crop production systems.

## Supporting information

S1 Table Number of fertigation events in the manual (M) and smart fertigation (S) treatments. The data for each replication and treatment are shown.
(DOCX)

S2 Table Canopy area of plants in the manual (M) and smart fertigation (S) treatments. The data are shown for different days in each replication and treatment during the experiment.
(DOCX)
