# Supplementary material for: Smart fertigation: Automated fertilizer application based on plant growth using digital sensors with IoT capability
Source: PLoS One. 2026 Sep 3;21(9):e0357482. doi: 10.1371/journal.pone.0357482 (PMC13541140; doi:10.1371/journal.pone.0357482)
Supplement: Supplementary file 1 [file pone.0357482.s001.docx]

Supplemental Table 1. Number of fertigation events in the manual (M) and smart fertigation (S) treatments. The data for each replication and treatment are shown.

| Replication | Treatment | Fertigation Events |
| --- | --- | --- |
| 1 | M | 6 |
| 1 | S | 4 |
| 2 | M | 6 |
| 2 | S | 1 |
| 3 | M | 6 |
| 3 | S | 1 |
| 4 | M | 6 |
| 4 | S | 0 |
