# Supplementary material for: Smart fertigation: Automated fertilizer application based on plant growth using digital sensors with IoT capability
Source: PLoS One. 2026 Sep 3;21(9):e0357482. doi: 10.1371/journal.pone.0357482 (PMC13541140; doi:10.1371/journal.pone.0357482)
Supplement: Supplementary file 2 [file pone.0357482.s002.docx]

Supplemental Table 2. Canopy area of plants in the manual (M) and smart fertigation (S) treatments. The data are shown for different days in each replication and treatment during the experiment.

| Replication | Treatment | Canopy Area (cm^2^) | | | | |
| --- | --- | --- | --- | --- | --- | --- |
|  |  | Day 4 | Day 7 | Day 10 | Day 13 | Day 16 |
| 1 | M | 97.94 | 173.68 | 270.42 | 428.77 | 772.01 |
| 1 | S | 89.47 | 128.07 | 214.26 | 338.25 | 663.02 |
| 2 | M | 62.74 | 126.33 | 162.12 | 324.52 | 645.52 |
| 2 | S | 64.59 | 112.97 | 189.11 | 360.48 | 628.2 |
| 3 | M | 77.07 | 129.88 | 207.26 | 378.65 | 776.57 |
| 3 | S | 65.59 | 116.77 | 197.93 | 368.85 | 835.16 |
| 4 | M | 73.03 | 150.38 | 257.2 | 501.11 | 935.73 |
| 4 | S | 89.69 | 168.81 | 305.69 | 578.88 | . ^1^ |
